# A Severe Episode of Hemolytic Anemia After Amoxicillin Exposure in A G6PD Deficient Patient

**DOI:** 10.26502/acmcr.96550068

**Published:** 2019-05-20

**Authors:** Carmelo J. Blanquicett, Tapasya Raavi, Stephanie M. Robert

**Affiliations:** 1 Department of Hematology and Medical Oncology, Moffitt Cancer Center, Tampa, FL, USA; 2 Department of Medicine, Division of General Medicine and Geriatrics, Emory University School of Medicine and Atlanta VA Medical Center, Atlanta, GA, USA; 3 Emory University Rollins School of Public Health, Atlanta, GA, USA; 4 Yale University School of Medicine, Department of Neurosurgery, New Haven, CT, USA

**Keywords:** Glucose-6-phosphate dehydrogenase, Hemolytic anemia, Enzyme deficiency, Amoxicillin

## Abstract

Glucose-6-phosphate dehydrogenase (G6PD) deficiency is the most common enzyme deficiency worldwide, with genetic variants resulting in a range of phenotypes that vary from asymptomatic to severe hemolysis. We report a case of severe hemolytic anemia in a G6PD deficient patient whose only known exposure was amoxicillin two weeks prior to his episode of severe hemolysis, for which he presented to our hospital. An extensive infectious and hematologic workup resulted negative with the exception of a positive G6PD deficiency result. Although rare, we suggest that the patient’s severe hemolytic anemia is possibly related to amoxicillin exposure.

## 1. Background

Glucose-6-phosphate dehydrogenase (G6PD) deficiency is the most common enzyme deficiency, with an estimated 400 million people affected worldwide [1]. There are several variants of the disease resulting in a wide spectrum of presentations ranging from asymptomatic to severe hemolysis. In the most prevalent types, the Mediterranean and the A- variant, hemolysis occurs after an exposure to oxidizing agents, typically antimalarial or sulfonamide drugs; infectious agents, such as Parvovirus, Cytomegalovirus (CMV), or Hepatitis. In this report, we present an intriguing case of severe hemolytic anemia in a 23 year old patient who was exposed to amoxicillin, ten days prior to presentation, and ultimately found to be G6PD deficient. Only two previous associations of severe hemolysis due to amoxicillin exposure have been reported [2, 3]. While rare, no infectious or alternative chemical exposure was found, and no alternative inciting event could be identified. We, therefore, propose that our patient’s presentation of G6PD-mediated hemolytic anemia is likely a result of amoxicillin exposure.

## 2. Case Presentation

A 23 year old, bi-racial (Hispanic-Caucasian) male was admitted to an outside hospital (OSH) with complaints of jaundice, generalized weakness, and vomiting. On admission, he was noted to have a hemoglobin (Hgb) level of 3.7 mg/dl (Reference Range (RR) 14–18 mg/dl), pancytopenia, and hyperbilirubinemia (total bilirubin 18.9; RR 0.3–1.2 mg/dl). The patient was admitted to the Intensive Care Unit (ICU) and received 13 units of blood during his admission. An extensive workup, including the Human Immunodeficiency Virus (HIV), Parvovirus B19, Cytomegalovirus (CMV), Epstein-Barr virus (EBV), hepatitis panel, and bone marrow biopsy was unrevealing; however, he slowly improved and was discharged home 2 weeks after admission with a Hgb of 8.0 mg/dl. Two days later, he developed non-radiating chest pain, prompting presented to our hospital, at which time he was found to have a Hgb of 8.4 mg/dl. Importantly, 10 days prior to admission to the OSH ICU, the patient had completed a 14day course of amoxicillin for an infection and subsequent removal of an ingrown toenail. On presentation, the patient denied fevers, chills, rigors, dyspnea, weight-loss, and lymphadenopathy. Review of symptoms was positive for dysuria since placement and subsequent removal of a Foley catheter at the OSH, as well as a mild headache. He reported that he was in his usual state of good health until approximately 1 week after completing the course of amoxicillin, when he began feeling unwell. Two to three days prior to initial admission, he experienced non-bilious, non-bloody emesis and nausea with loose stools. On the morning of admission, his urine became dark and he appeared jaundiced, which prompted his parents to bring him to the OSH’s emergency room. His past medical history was notable for paranoid schizophrenia, for which he was being treated with clonazepam 0.5 mg po BID, quetiapine 25 mg po TID PRN, sertraline 200 mg QD, and ziprasidone 80 mg BID, all of which were discontinued on admission to the OSH. Past surgical history was unremarkable. The patient had no known allergies and denied illicit drug use, tobacco, and alcohol use. Further, the patient was sexually inactive. He had a family history remarkable for unexplained blood clots on his agnate grandmother’s side. A couple of years prior to presentation he had been stationed in Afghanistan without notable illness. On arrival to our medical unit, physical examination revealed a blood pressure of 125/75 mm Hg, a heart rate of 100 beats per minute (bpm), a respiration rate of 18 breaths per minute, and a temperature of 98.1°F. The patient was alert and oriented, but anxious in appearance. The head-eyes-ears-nose-throat (HEENT) exam revealed scleral icterus, and the cardiopulmonary exam was within normal limits, except for borderline tachycardia. Abdominal exam was notable for diffuse tenderness to deep palpation and splenomegaly without hepatomegaly. No focal deficits were observed on neurological exam, cranial nerves were intact, and no meningismus was appreciated.

**Figure 1:**
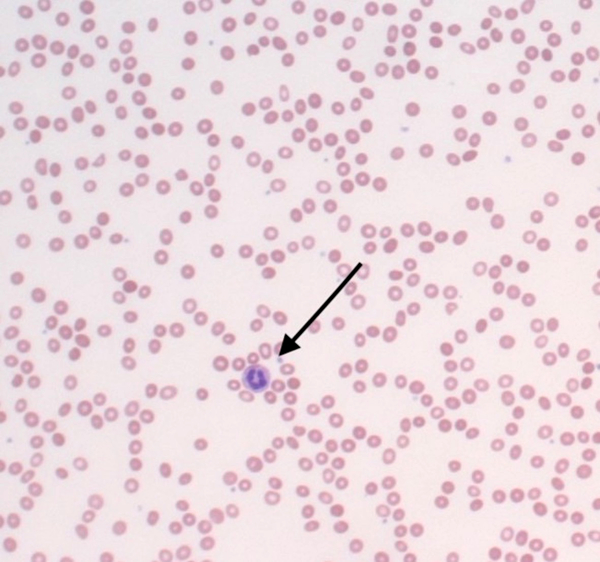
Microcytic, normochromic peripheral smear with moderate anisopoikilocytosis. Neutrophils (arrow) are mildly decreased in number, with unremarkable morphology.

Laboratory findings demonstrated pancytopenia with a low white blood cell (WBC) count of 2.3 thousand/cmm (RR 4.5–11.0 thousand/cmm) and neutrophilic predominance of 68% (RR 37–77%), thrombocytopenia with a platelet count of 105 thousand/cmm (RR 150–350 thousand/cmm), and a hemoglobin and hematocrit that were 8.2 mg/dl and 24%, respectively (Table 1). Absolute reticulocyte count was low at 0.0134 million/cmm (Table 1), with a relative value that was at the low end of normal at 0.4% (RR 0.4–1.9%). A peripheral blood smear was microcytic with central pallor, as shown in Figure 1. Lymphocyte markers assessed by flow cytometry showed no evidence of clonal lymphoid expansion. Erythropoietin was high, osmotic fragility testing and direct red blood cell antibody assay were negative (Table 2). A complete biochemistry panel, including cardiac markers (Troponin, creatine kinasemuscle/brain (CK-MB), creatine kinase (CK)), was unremarkable. Urinalysis (UA) was revealing for 1+ protein, elevated urobilinogen (4.0 mg/dl; RR 0.1–1.0 mg/dl), 2+ leukocyte esterase and positive nitrites; urine microscopy showed 29 RBC/high-powered field (hpf) and 185 WBC/hpf. Urine drug screen was negative. Liver function tests revealed an elevated aspartate aminotransferase (AST)/ serum glutamic oxaloacetic transaminase (SGOT) and alanine aminotransferase (ALT)/ serum glutamate-pyruvate transaminase (SGPT), with alkaline phosphatase within normal limits (Table 3). International normalized ratio (INR) was 1.21 and total bilirubin was 4.05 mg/dl (RR 0.31.2 mg/dl) with a direct bilirubin of 1.1 mg/dl (RR 0.00–0.34 mg/dl), as shown in Tables 2 and 3. Lactate dehydrogenase (LDH) resulted 1080 U/L (RR 135–225 U/L); D dimer was 300; total protein was within normal limits, and C-reactive protein (CRP) was unremarkable (Table 2). Both ferritin and fibrinogen were elevated at 911 ng/ml (RR 10–385 ng/ml) and 426 mg/dl (RR 198–450 mg/dl), respectively (Tables 2 and 3).

A chest radiograph proved to be normal and abdominal CT showed marked splenomegaly (not shown). CD 4 counts and percentages were normal. Cold agglutinins were negative, as was a COOMBS test. Donnath Landsteiner test was also negative (Table 2). The infectious workup was grossly negative (Table 4). The only source of infection found was the patient’s UA, as above, revealing for a urinary tract infection (UTI) that grew Klebsiella *pneumoniae,* which was attributed to an indwelling Foley catheter during his 7-day stay at the OSH ICU. Rheumatology tests were also negative, including Antineutrophil Cytoplasmic Antibodies (ANCA) and Rheumatoid factor (Table 2). Subsequently, a bone marrow biopsy was performed, showing a hypercellular marrow (90%) with remarkable erythroid hyperplasia and left shift, mild megakaryocytic hyperplasia without dysplasia or atypia, with no atypical cell infiltrate identified. A repeat reticulocyte count, 11 days after admission, resulted as an absolute value of 0.0261 mill/cmm (RR 0.026–0.095 mill/cmm) and a relative value of 0.9% (RR 0.4–1.9%). G6PD testing by enzymatic methods used for definitive diagnosis [4] yielded positive results for deficiency. The patient’s transfusion requirements diminished, as he only required an additional 2 units of packed red blood cells and was treated for his UTI with ciprofloxacin.

The patient was discharged on a steroid taper and folic acid. Two months after discharge, hemoglobin normalized to 15.5 g/dl. He continued to improve steadily at home and three months after initial symptoms, he fully recovered and returned to his prior state of health and activity level. He has not had any further hemolytic events, however, he received follow-up in our Hematology/Oncology clinic.

## 3. Discussion

G6PD is the most common enzyme deficiency worldwide, with a global prevalence of 4.9% [1]. For the majority of people affected, G6PD deficiency is asymptomatic; however, upon exposure to oxidizing or infectious agents, hemolysis can occur, leading to precipitous drops in blood counts requiring hospital admission and blood transfusion. The patient described in this case report presented with severe hemolysis, requiring multiple administrations of blood products, in addition to an extended hospital admission due to ongoing hemolysis. After extensive laboratory testing investigation, G6PD deficiency was revealed. Interestingly, the only new exposure prior to the onset of hemolysis was to amoxicillin, which albeit rare, has previously been reported to induce hemolysis in two prior published cases [2, 3], with one being in a G6PI deficient patient [2]. Although our patient was 10 days post-exposure at initial admission, he reported a 1-week history of symptoms, which fits with the reported timeline in these previously-published cases.

The reticulocyte count was an unexpected finding in this patient. In conditions of massive hemolysis, an elevated reticulocyte count would be expected in a normally functioning bone marrow. In this patient, however, lab tests revealed a low reticulocyte count at admission. Bone marrow biopsy revealed a hyper-cellular bone marrow, suggesting either a response to hemolysis, or bone marrow involvement, which was ruled out with flow cytometry methods. A repeat reticulocyte count 11 days after presentation indicated a mild improvement, but the response remained suboptimal. Aplastic crisis has been previously reported in G6PD deficient patients, however, these were found to be a result of inciting viral infections such as CMV [5] and Parvovirus [6], which were not detected in our patient. Furthermore, splenomegaly has been associated with ongoing hemolysis in G6PD deficient patients and may explain the lack of reticulocytosis due to splenic sequestration and subsequent destruction.

The extended duration of chronic ongoing hemolysis was similar to that seen in the autoimmune hemolytic anemias. However, arguing against this etiology was a negative Coombs test, as well as a high bilirubinemia, which is not typically seen in immune-mediated reactions. After exhausting our investigation for potential triggers of hemolysis in G6PD deficient patients, no other alternate source accounts for our findings. In addition to inducing hemolysis in G6PD deficient patients, amoxicillin and amoxicillin/clavulanic acid combinations have both been reported to cause neutropenia and pancytopenia in non-G6PD deficient patients being treated for a variety of infections [7–9]. Further, there are 97 reports of bone marrow failure associated with amoxicillin that were reported to the U.S. Food and Drug Administration (FDA) [10]. Additionally, there are many reports of the combination amoxicillin/clavulanic acid antibiotic therapy causing hepatotoxicity [11] and some reports of amoxicillin alone causing hepatitis [12,13], as was observed in our patient.

Oxidative stress is a known mechanism of drug-induced hemolytic episodes in G6PD deficient patients [14–16]. G6PD protects cells from oxidative stress by playing a role in the reduction of glutathione, an important antioxidant and an essential component required for the maintenance of the normal RBC structure [17]. Bactericidal antibiotics, including β-lactams such as amoxicillin, have been shown to cause mitochondrial dysfunction and reactive oxygen species (ROS), resulting in oxidative damage [18]. Due to the late presentation of the patient to our hospital, direct confirmation of amoxicillin-induced hemolysis was not possible; however, our findings strongly suggest that this patient’s severe hemolytic anemia was possibly associated with amoxicillin exposure. Oxidative stress (potentially as a result of amoxicillin exposure), which is a known initiator of hemolysis in G6PD deficient patients, may have been severe enough to produce significant, ongoing hemolysis, as well as hepatotoxicity and subsequent splenomegaly in this case presentation. It would be of particular interest to perform genotyping testing of G6PD deficient patients to define particular G6PD variants that could determine which patients would be especially susceptible to amoxicillin-triggered hemolysis. New insights into G6PD polymorphisms may provide some clarification and better allude to the potential susceptibility of those patients that would respond with a severe hemolysis after amoxicillin exposure.

In conclusion, we describe an unusual presentation of severe hemolytic anemia, likely related to amoxicillin exposure in a G6PD deficient patient. These findings, in conjunction with previously-published cases, highlight the need to consider amoxicillin as a cause for the presenting symptoms described in this case report. Increased awareness of such potential would warrant caution when prescribing amoxicillin to G6PD deficient patients and may argue for further characterization of the precise G6PD variant, once a deficiency has been established.

## Figures and Tables

**Table 1:** Complete Blood Count.

Compound	Sample	Result	Reference Range	Units
Hemoglobin (Hgb)	Blood	8.2 (L)	14–18	mg/dl
Hematocrit (Hct)	Blood	24 (L)	40–54	%
Mean Corpuscular Value (MCV)	Blood	78.9 (L)	80–96	fL
White Blood Cell (WBC)	Blood	2.3 (L)	4.5–11.0	thousand/cmm
Red Blood Cells	Blood	3.04 (L)	4.6–6.2	million/cmm
Platelets	Blood	105 (L)	150–350	thousand/cmm
Reticulocyte Absolute Count	Blood	0.0134 (L)	0.026–0.095	million/cmm
Reticulocyte Relative Value	Blood	0.4	0.4–1.9	%

**Table 2:** Hematologic and Rheumatologic Labs.

Compound	Sample	Result	Reference Range	Units
**Hematologic Labs**				
CD4	Blood	65 (H); 275 (L)	30–61; 490–1740	cells/µl
CD8	Blood	19; 79 (L)	12–42; 180–1170	cells/µl
CD4/CD8 Ratio	Blood	3.48	0.86–5.00	-
Complement, CH50	Serum	54	31–60	U/ml
Cold Agglutinins	Serum	Negative	Negative	-
Direct RBC Ab Assay	Blood	Negative	Negative	-
COOMBS test	Blood	Negative	Negative	-
Donate Landsteiner	Flexitest, Serum	Negative	Negative	-
Erythropoietin	Serum	225.9 (H)	2.6–18.5	mIU/ml
D-Dimer	Plasma	300 (H)	< 234	ng/ml FEU
Haptoglobin	Serum	< 15 (L)	43–212	mg/dl
Lactate Dehydrogenase (LDH)	Serum	1080 (H)	135–225	U/L
International Normalized Ratio (INR)	Plasma	1.21	0.83–1.26	-
Ceruloplasmin	Serum	30	18–36	mg/dl
Fibrinogen	Plasma	426	198–450	mg/dl
Hemosiderin	Urine	Negative	Negative	-
Vitamin B12	Serum	572.8	200–900	pg/ml
Osmotic fragility test	Blood	Normal	Normal	%
**Rheumatologic Labs**				
Anti-Neutrophil cytoplasmic Antibody (ANCA)	Serum	Negative	Negative	-
Rheumatoid Factor (RF)	Serum	5	<= 14	IU/ml
Cyclic Citrullinated Peptide (CCP) Antibody	Serum	< 16	<= 20	U
Sjogren’s Antibody (SSA)	Serum	< 1.0	<= 1.0	U
Erythrocyte Sedimentation Rate (ESR)	Blood	60 (H)	0–15	mm/hr
C-Reactive Protein (CRP)	Serum	3.38	< 0.8	mg/dl
Sjogren’s Antibody (SSB)	Serum	< 1.0	<= 1.0	U

**Table 3:** Liver Function Tests and Iron Studies.

Compound	Sample	Result	Reference Range	Units
**Liver Function tests**				
Aspartate Aminotransferase (AST)	Serum	84 (H)	15–46	U/L
Alanine Aminotransferase (ALT)	Serum	89 (H)	11–66	U/L
Alkaline Phosphatase	Serum	51	38–126	U/L
Total Bilirubin	Serum	4.03 (H)	0.3–1.2	mg/dl
Direct Bilirubin	Serum	1.1 (H)	0.00–0.34	mg/dl
Total Protein	Serum	6.7	6.0–8.3	g/dl
Albumin	Serum	4.3	3.5–5.0	g/dl
**Iron Studies**				
Iron	Serum	83	49–181	ug/dl
Folate	Serum	9.04	2.5–17	ng/ml
Iron Saturation	Serum	32.0c	20–50	%
Total Iron Binding Capacity (TIBC)	Serum	262c	250–450	ug/dl
Ferritin	Serum	911 (H)	10–385	ng/ml

**Table 4:** Infectious Disease Labs.

Infection Cause	Sample	Result	Reference Range	Units
Adenovirus	Serum	Negative	Negative	-
Epstein-Barr Virus (EBV)	Serum	Negative	Negative	-
Epstein-Barr Virus (EBV)	Plasma	< 200	<= 200	copies/ml
Cytomegalovirus (CMV)	Serum	Not Detected	Not Detected	-
Parvovirus B19	Serum	Not Detected	Not Detected	-
Parvovirus B19	Bone Marrow	Not Detected	Not Detected	-
Enterovirus	Plasma	Not Detected	Not Detected	-
HIV	Serum	Nonreactive	Nonreactive	-
Rapid Plasma Reagin (RPR)	Serum	Nonreactive	Nonreactive	-
Hepatitis A	Serum	Nonreactive	Nonreactive	-
Hepatitis B	Serum	< 20	<= 20	IU/ml
Hepatitis C	Serum	Nonreactive	Nonreactive	-
Toxoplasma gondii	Serum	Negative	Negative	-
Histoplasma capsulatum	Urine	Negative	Negative	-
Cryptococcus neoformans	Serum	Negative	Negative	-
Ehrlichia chaffeensis	Serum	Not Detected	Not Detected	-
Mycobacterium tuberculosis	Blood	Negative	Negative	-
Borrelia burgdorferi	Serum	Negative	Negative	-
Rickettsia rickettsii	Serum	Not Detected	Not Detected	copies/ml
Coccidioides immitis	Plasma	Negative	Negative	-
Bartonella henselae	Serum	Negative	Negative	-
Leptospira interrogans	Serum	Negative	Negative	-
Haemophilus influenzae	Serum	Negative	Negative	µg/mL
Neisseria gonorrhoeae	Urine	Negative	Negative	-
Chlamydia trachomatis	Urine	Negative	Negative	-
Eosinophilia	Urine	0	0–1	%
Urine culture	Urine	Positive-Klebsiella pneumoniae	no growth	cfu/ml
